# Anaphylaxis in Elderly Patients—Data From the European Anaphylaxis Registry

**DOI:** 10.3389/fimmu.2019.00750

**Published:** 2019-04-24

**Authors:** Stefanie Aurich, Sabine Dölle-Bierke, Wojciech Francuzik, Maria Beatrice Bilo, George Christoff, Montserrat Fernandez-Rivas, Thomas Hawranek, Claudia Pföhler, Iwona Poziomkowska-Gȩsicka, Jean-Marie Renaudin, Eva Oppel, Kathrin Scherer, Regina Treudler, Margitta Worm

**Affiliations:** ^1^Department of Dermatology, Venereology and Allergology, LICA-Comprehensive Allergy Center, University Hospital, Leipzig, Germany; ^2^Division of Allergology and Immunology, Department of Dermatology, Venereology and Allergology, Charité-Universitätsmedizin Berlin, Berlin Institute of Health, Corporate Member of Freie Universität Berlin, Humboldt-Universität zu Berlin, Berlin, Germany; ^3^Allergy Unit, Department of Internal Medicine, University Hospital Ospedali Riuniti, Ancona, Italy; ^4^Faculty of Public Health, Medical University-Sofia, Sofia, Bulgaria; ^5^Allergy Outpatient Unit, Acibadem CityClinic, Medical Centre Tokuda, Sofia, Bulgaria; ^6^Allergy Department, Hospital Clinico San Carlos, Madrid, Spain; ^7^Department of Dermatology, University Hospital of the Paracelsus Private Medical University, Salzburg, Austria; ^8^Department of Dermatology, Venereology and Allergology, Saarland University Medical Center, Homburg, Germany; ^9^Clinical Allergology Department, Pomeranian Medical University in Szczecin, Szczecin, Poland; ^10^Presidency, Allergy Vigilance Network, Vandoeuvre les Nancy, France; ^11^Department of Dermatology and Allergy, University Hospital (LMU), Munich, Germany; ^12^Division of Allergy, Department of Dermatology, University of Basel, Basel, Switzerland

**Keywords:** anaphylaxis, elderly, insect venom, drug hypersensitivity, adrenaline, hospitalization, loss of consciousness

## Abstract

**Background:** Elicitors and symptoms of anaphylaxis are age dependent. However, little is known about typical features of anaphylaxis in patients aged 65 years or more.

**Methods:** The data from the Network for Online Registration of Anaphylaxis (NORA) considering patients aged ≥65 (elderly) in comparison to data from adults (18–64 years) regarding elicitors, symptoms, comorbidities, and treatment measures were analyzed.

**Results:** We identified 1,123 elderly anaphylactic patients. Insect venoms were the most frequent elicitor in this group (*p* < 0.001), followed by drugs like analgesics and antibiotics. Food allergens elicited less frequently anaphylaxis (*p* < 0.001). Skin symptoms occurred less frequently in elderly patients (77%, *p* < 0.001). The clinical symptoms were more severe in the elderly (51% experiencing grade III/IV reactions), in particular when skin symptoms (*p* < 0.001) were absent. Most strikingly, a loss of consciousness (33%, *p* < 0.001) and preexisting cardiovascular comorbidity (59%, *p* < 0.001) were more prevalent in the elderly. Finally, adrenaline was used in 30% of the elderly (vs. 26% in the comparator group, *p* < 0.001) and hospitalization was more often required (60 vs. 50%, *p* < 0.001).

**Discussion and Conclusion:** Anaphylaxis in the elderly is often caused by insect venoms and drugs. These patients suffer more often from cardiovascular symptoms, receive more frequently adrenaline and require more often hospitalization. The data indicate that anaphylaxis in the elderly tends to be more frequently life threatening and patients require intensified medical intervention. The data support the need to recognize anaphylaxis in this patient group, which is prone to be at a higher risk for a fatal outcome.

## Introduction

The incidence of anaphylaxis, which may occur at any age, is rising in Western countries (1, 2). While life expectancy is increasing, little is known about the characteristics of anaphylaxis in elderly patients (3). Existing data indicate age dependent differences with regard to elicitors, cofactors, and symptoms of anaphylaxis (4).

Overall, elderly patients have a higher risk for severe and fatal reactions (5).

The main elicitors of anaphylaxis are insect venoms, drugs and food items (6). While food items are the most frequent elicitors in children and young adults (7), drug anaphylaxis is more common in elderly patients (8). Patients above 65 were reported to be prone to develop cardiovascular symptoms more frequently (9). It is unknown whether this is due to the increased prevalence of cardiovascular diseases in this age group leading to limited cardiovascular compensation mechanisms (10), use of cardiovascular drugs (5), or both. Cofactors like exercise, drugs, alcohol, and stress are supposed to reduce the threshold of allergic reactions (11).

Recommendations for the emergency treatment of anaphylaxis are similar for all age groups and are supported by current guidelines (12). Some considerations and adaptions should be made in elderly patients. Although, the administration of adrenaline in anaphylactic patients with known or suspected cardiovascular diseases is not contraindicated, it might sometimes cause difficulties due to an increased coronary blood flow e.g., in patients with acute coronary syndrome (10). However, current guidelines clearly state that the benefit of adrenaline usage outweighs its risks through beneficial effects even in suspected anaphylaxis (12).

In this study, we analyzed data from adult patients differentiated by age from the European Anaphylaxis Registry registered between 2007 and 2017. The aim was to better understand anaphylaxis in patients ≥65 years. Data regarding elicitors, symptoms, comorbidities, and emergency treatment were considered.

## Methods

### Setting and Design

The European Anaphylaxis Registry collects information on anaphylactic reactions through a web based electronic data capture system as described previously (6). Participation of study centers is voluntary. Data for the current analysis were provided by tertiary referral centers specialized in allergology and/or dermatology in Germany, Switzerland, France, Austria, Spain, Italy, Bulgaria, and Poland. The study was approved by the Ethics Committee at Charité-Universitätsmedizin Berlin (the coordinating center) and by the local Ethics Committees.

### Data Source and Handling

After completion of diagnostics, patients' data were retrieved from medical treatment, laboratory measurements, and emergency protocols as available. Using a pseudonym, the data were entered by trained study personnel into an online questionnaire in each study center. The questionnaire and data entry is described elsewhere (7). Data collected from July 2007 to March 2017 were included.

### Variables

Age at reaction was categorized in two groups: patients aged 18–64 years (adults), and patients 65 years and older (elderly). The gathered variables are described elsewhere (7).

### Statistical Analysis

The analysis of the data was carried out using R. The Shapiro-Wilk's test was used to assess the normality of distribution of interval variables. Interval variables were expressed as mean ± standard deviation. Distribution of categorical variables were expressed as percentages. Differences in categorical variables were tested using pairwise Chi^2^ tests with Holm's correction for multiple comparisons. The values *P* < 0.05 were considered statistically significant.

## Results

The European Anaphylaxis Registry collected data of 10,203 cases between July 2007 and March 2017. Six thousand eight hundred ninety-one patients met the criteria of Ring and Messmer ≥grade II and were aged ≥18 years. The cases were registered from 78 study centers in seven countries: Germany (*n* = 4,474), Switzerland (*n* = 777), France (*n* = 529), Austria (*n* = 461), Spain (*n* = 267), Italy (*n* = 188), Bulgaria (*n* = 100), and Poland (*n* = 95).

**Figure 1:**
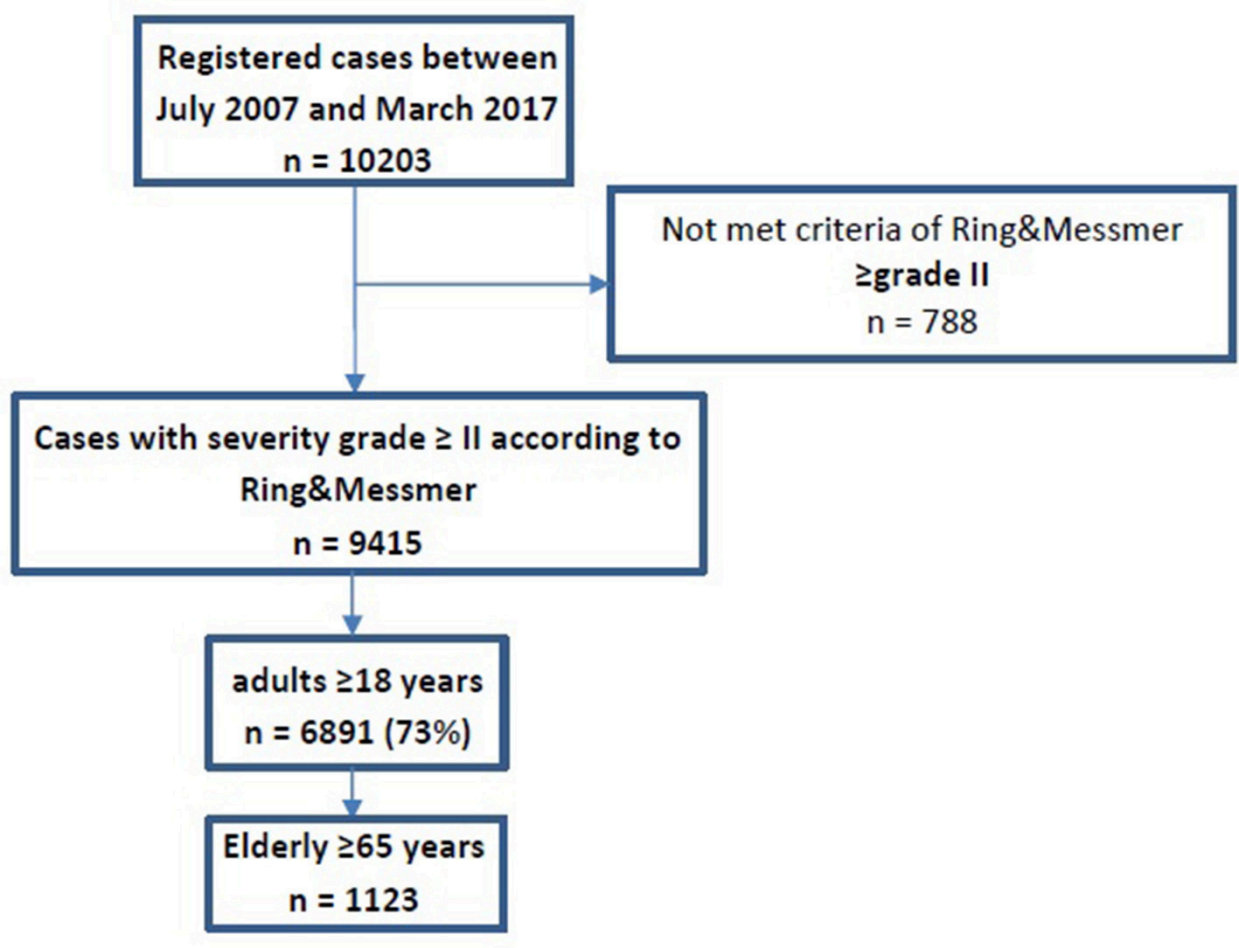
Flow chart of study cohort.

**Figure 2:**
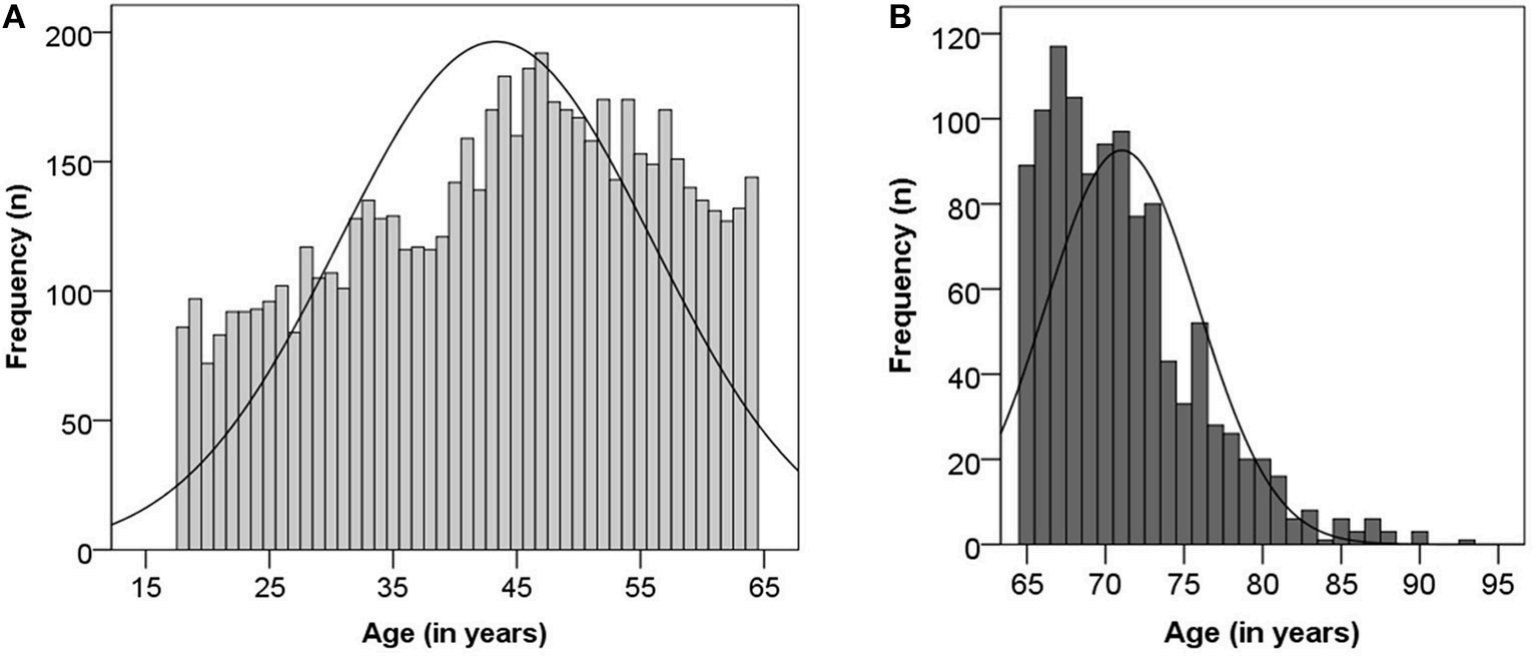
Age distribution among adults **(A)** and the elderly **(B)**.

Of the 6,891 cases, 5,768 were adults younger than 65 years and 1,123 were ≥65 years old (Figures 1, 2). The median age in elderly patients was 70 (65–93) years and 95% of the cases are between 65 and 80 years (Figure 2B). In general, both adult groups comprised more females than males, but the elderly included a higher percentage of male patients in comparison to the comparator adult group (*p* = 0.005, Table 1).

**Table 1:** Characteristics of the elderly in comparison to the adult group.

	**Elderly (≥65 years)**	**Adults (18–64 years)**	
	***n* (%)**	***n* (%)**	***Chi^**2**^ test***
**TOTAL**	1,123 (16)	5,768 (84)	
Median age in years (min–max)	70 (65–93)	45 (18–64)	
Male	517 (46)	2,394 (42)	*p = 0.005*
**COMORBIDITIES**			
Atopic	190 (18)	1,628 (30)	*p < 0.001*
Cardiovascular	641 (60)	960 (18)	*p < 0.001*
Mastocytosis	39 (4)	136 (3)	*NS (p = 0.051)*
Thyroid	162 (15)	476 (9)	*p < 0.001*
Urticaria	14 (1)	104 (2)	*NS*
Malignant disease	63 (6)	97 (2)	*p < 0.001*
**COFACTORS**			
Overall	802 (71)	2,897 (54)	*p < 0.001*
Drugs^#^	614 (57)	991 (18)	*p < 0.001*
Physical exercise	267 (27)	1,609 (31)	*p = 0.02*
Psychological stress	84 (8)	418 (7)	*NS*
Alcohol	48 (5)	326 (7)	*NS (p = 0.051)*
Acute Infection	33 (3)	177 (3)	*NS*
**SEVERITY ACCORDING TO RING&MESSMER**			
Grade II	550 (49)	3,505 (61)	*p < 0.001*
Grade III	525 (47)	2,105 (37)	*p < 0.001*
Grade IV	48 (4)	158 (3)	*p = 0.023*

*In total 6,891 cases ≥18 years old were analyzable until March 2017. Statistical calculations were performed with chi^2^ test including post-hoc corrections. Significant differences were given in comparison to adults between 18 and 64 years of age*.

# *Drugs counted as cofactors were ace-inhibitor, AT-2-antagonist, beta-blocker, acetylcholine, and proton pump inhibitor*.

The recent medical history in elderly patients is characterized by significantly more frequent cardiovascular diseases, thyroid and malignant diseases than in younger adults. In contrast, in the younger adult group, atopic diseases were significantly more common than in the elderly (*p* < 0.001).

Potential cofactors of any type (Table 1) were significantly more prevalent in elderly patients (*p* < 0.001) and there was a considerable association between the age and the concomitant drug intake, under which ace inhibitor, AT-2 antagonist, beta-blocker, acetylcholine, and proton pump inhibitor were taken into account (Table 1; *p* < 0.001). Twenty-eight percentage of elderly patients reported a previous allergic reaction to the same elicitor. Of these 75% reported a previous milder reaction, 19% had a similar or even more severe reaction (data not shown).

### Insect Venom and Drugs Are Main Causes of Anaphylaxis in the Elderly

Elicitors were more frequently identified in the elderly (79%) than in the younger adult group (74%; *p* < 0.001; Table 2). Insect venom anaphylaxis was most prevalent in elderly patients (*n* = 633; 56%; *p* < 0.001), with yellow jacket (*n* = 451, 71%) being the cause in the majority of cases. Drugs were the second main causative agent of anaphylaxis across adults again being more prevalent in the elderly (25%; *p* = 0.009). Analgesics [metamizole (*n* = 19; 7%), diclofenac (*n* = 45; 16%), ibuprofen (*n* = 17; 6%)] and antibiotics [penicillin (*n* = 23; 8%), cephalosporins (*n* = 30; 11%), gyrase inhibitors/ quinolones (*n* = 14; 5%)] were the most frequent single elicitors of drug anaphylaxis in the elderly. Food was a predominant elicitor in adults (22%, *p* < 0.001; Table 2). In the elderly only 11% of reactions were caused by food, with wheat (14%) and hazelnut (13%) as leading food allergens.

**Table 2:** Elicitor profile in the elderly in comparison to the younger adult group.

	**Elderly (≥65 years)**	**Adults (18–64 years)**	
	***n* (%)**	***n* (%)**	***Chi^**2**^ test***
**TOTAL**	1,123 (16%)	5,768 (84%)	
Elicitor known	883 (79)	4,242 (73)	*p < 0.001*
**INSECTS**	633 (56)	2,708 (47)	*p* < 0.001
Yellow jacket	451 (71)	1,936 (72)	*NS*
Bee	97 (15)	512 (19)	*NS*
Hornet	56 (9)	153 (6)	*p* < 0.001
**DRUGS**	285 (25)	1,257 (22)	*p = 0.009*
Analgesics	103 (36)	354 (36)	*NS*
Antibiotics	74 (26)	354 (28)	*NS*
Local anesthetics	17 (6)	106 (8)	*NS*
x-ray (contrast agent)	22 (8)	56 (4)	*P = 0.010*
PPI	6 (2)	30 (2)	*NS*
Cardiovascular drugs	5 (1.8)	8 (0.6)	*NS*
**FOOD**	122 (11)	1,254 (22)	*p < 0.001*
Wheat	17 (14)	177 (14)	*NS*
Hazelnut	16 (13)	84 (7)	*p = 0.015*
Shellfish	15 (12)	123 (10)	*NS*
Celery	7 (6)	74 (6)	*NS*
Soy	6 (5)	78 (6)	*NS*
Peanut	1 (0.8)	68 (5)	*p = 0.045*
**IMMUNOTHERAPY (SIT)**	5 (0.4)	63 (1.1)	*p = 0.006*

*The elicitor was not specified in 423 (6%) patients. Rare elicitors are not reported in detail*.

*Statistical analysis was performed with Chi^2^ test including post-hoc corrections. Significant differences were given in comparison to adults between 18 and 64 years of age. Food items reported < 10 times in the whole study are not listed in detail. PPI, proton pump inhibitor*.

### Cardiovascular Symptoms Predominate in the Elderly

**Figure 3:**
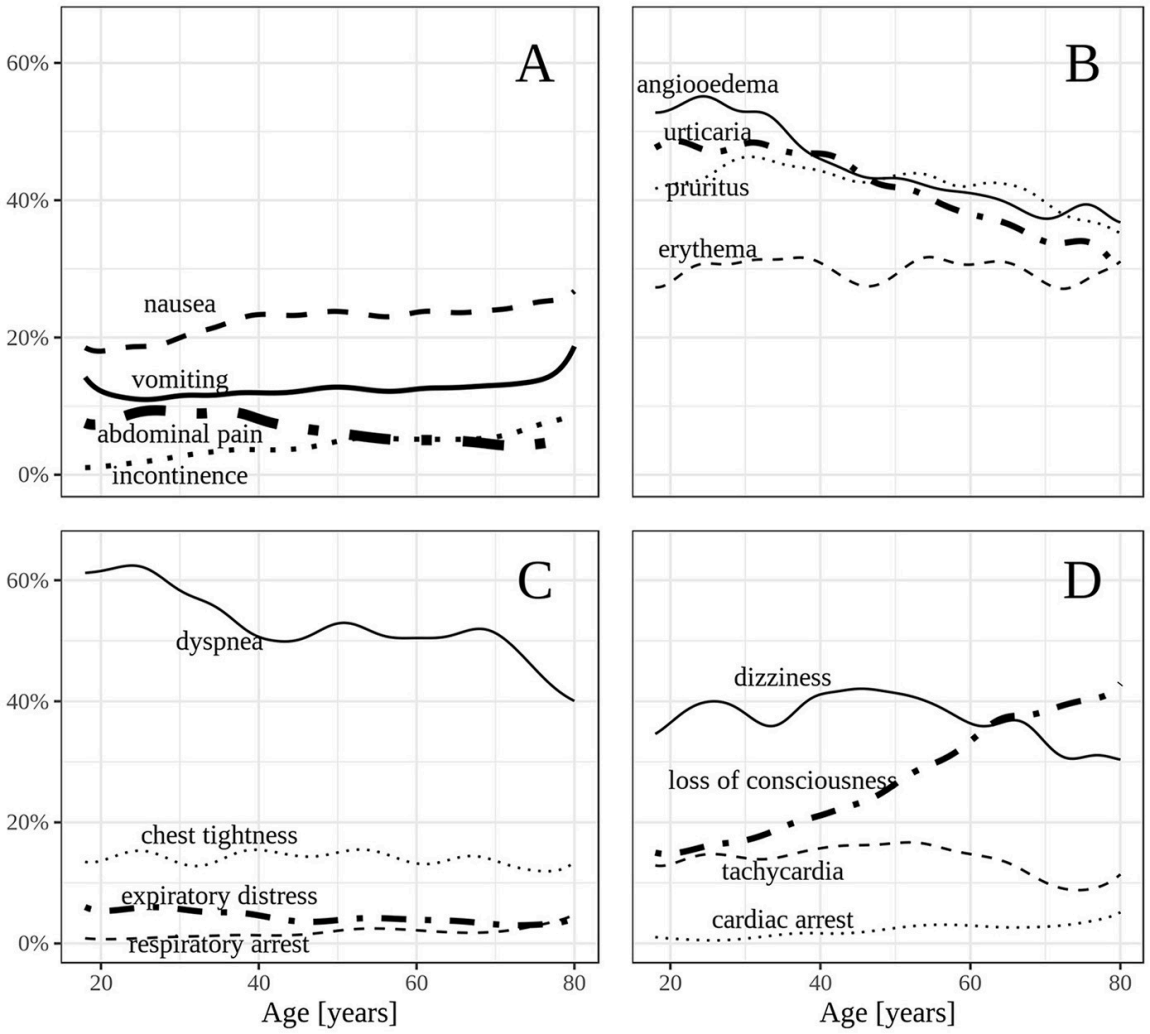
**(A–D)** Frequency of selected symptoms according to age in adults.

Cardiovascular symptoms occurred frequently in elderly patients (80% compared to adults 75%; *p* < 0.001). A major cardiovascular symptom was loss of consciousness (adults: 20%; elderly 33%, *p* < 0.001) while dizziness and tachycardia were more prevalent in adults (Figure 3D). Cardiac arrest occurred in 153 cases (3% in elderly vs. 2% in adults). The skin was the most frequently involved organ system of all affected patients. However, there was a shift showing that elderly patients are less frequently affected (77%) in comparison to younger adults (83%; *p* < 0.001; Figure 3A). The severity of the reactions in elderly patients without skin symptoms was increased in comparison to the corresponding group of adults not suffering from skin symptoms (*p* < 0.001, data not shown). Gastrointestinal symptoms occurred in a similar proportion in both adult groups (Figure 3B). The respiratory system was less frequently affected in the elderly (63% compared to adults 70%; *p* < 0.001), especially dyspnea (adults: 55%; elderly 51%; *p* < 0.001; Figure 3C). Severe anaphylactic reactions including grade III (47%) and grade IV (4%) anaphylaxis were more prevalent in the elderly (Table 1).

### Increased Administration of Adrenaline in the Elderly

**Figure 4:**
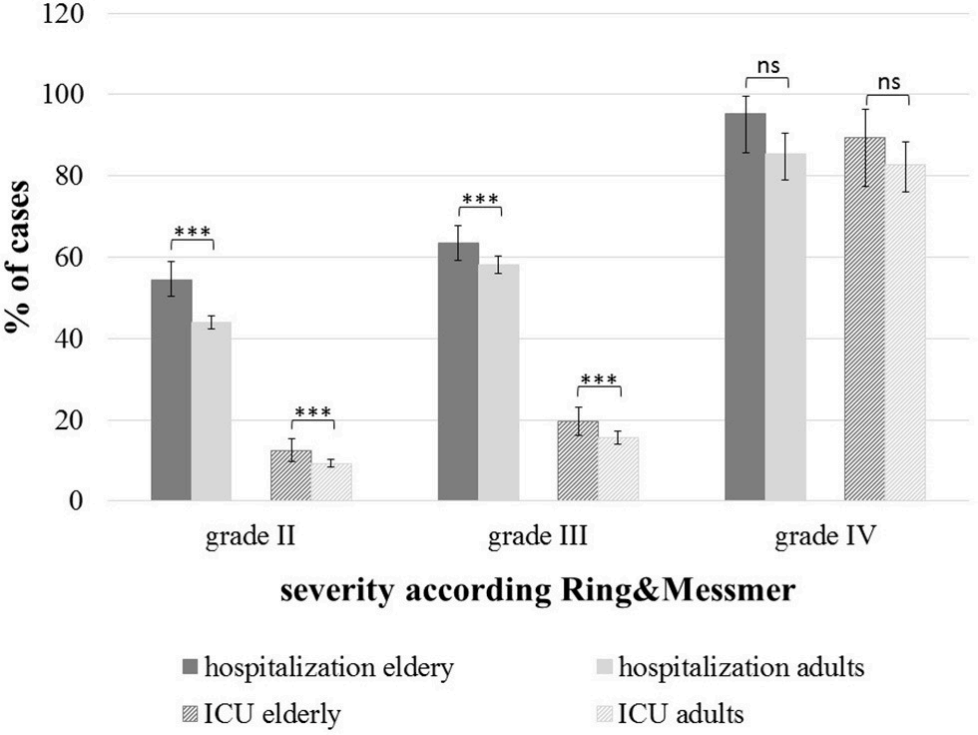
Hospitalization and intensive care unit admissions across the severity grades according to Ring & Messmer; elderly (*n* = 1,123) and adults (*n* = 5,768); ^***^p < 0.001, ns, not significant.

First-line treatment was carried out by emergency physicians in 5,481 (92%) of 5,971 patients who received first-line treatment. The administration rate of adrenaline by professionals in elderly patients was 30 and 26% in younger adults (Figure S1, across all severity grades; *p* = 0.043). Hospitalization was required in 60 and 19% of elderly patients were treated in an intensive care unit (ICU) (Figure 4). Regardless the severity grade, the hospitalization and the ICU admission rates were higher in the elderly (*p* < 0.01).

## Discussion

### Key Results

This is the first large-scale analysis of anaphylactic patients including 1,123 cases aged 65 years or more. The European Anaphylaxis Registry covers a heterogenic sample of patients across all age groups and a wide range of elicitors, from different European countries (6, 13).

The most frequent elicitor in both adult groups was insect venom, but elderly patients had significantly more insect elicited cases than younger adults (56 vs. 47%). The data indicate that the clinical reactivity to insect venoms increases with age, although the individual exposure to insect stings might be comparable among adults, whereas one could speculate that retired elderly spend more time outdoor, e.g., in the garden. However, supposing comparable exposure, one reason for this observation might be the higher frequency of comorbidities, which may enhance the clinical response pattern particularly in patients with preexisting cardiovascular diseases increasing the risks for severe cardiovascular symptoms like tachyarrhythmia or syncope.

Moreover, we identified drugs as frequent elicitors in the elderly. This finding is in line with previous data from the registry and most likely associated with a higher drug intake in this patient group (6, 14). Also, drug interaction maybe relevant in this context. Another previous study analyzing patients ≥71 years identified males to be predisposed to develop insect sting anaphylaxis whereas females suffered more often from drug anaphylaxis (15).

Furthermore, the distribution of elicitors differ by country (6). This observation is also reflected in the elderly patient group from different countries, e.g., drugs were the most frequent elicitors in Bulgaria and Spain and not insect venoms (data not shown).

The skin is the most frequently involved organ system in anaphylaxis as shown by many different studies including our own data (6). Most interestingly, the occurrence of skin symptoms in the elderly was significantly reduced compared to the younger adults. Moreover, this lack of skin symptoms was associated with more severe anaphylaxis in the group of the elderly patients. This phenomenon has been described previously and been attributed to the fast development of a severe circulatory impairment (16). Another assumption regarding the lack of skin symptoms and the onset of a severe reaction might be a delay of the diagnosis. A lack of skin symptoms leads to a significant reduced identification rate of anaphylaxis and consequently to delayed therapy (17). However, this would still not explain why skin symptoms are less frequent in this patient group, but may also be attributed to a decreased vascularization of the skin in higher age groups. A final hypothesis might be the intake of drugs affecting skin reactivity like betablockers or neuroleptics.

Cardiovascular manifestations of anaphylaxis occurred more frequently in our elderly patient cohort. Most interestingly, loss of consciousness was the predominant symptom in this patient group and has been observed by us in a previous analysis (18). Considering the overall adult cohort, most patients suffering from cardiovascular symptoms presented with dizziness and hypotension.

Adrenaline is the first treatment of choice in anaphylaxis and recommended by several international guidelines (12). However, data from others as well as our group indicate that most patients are not treated according to guidelines. By contrast, professional first-line treatment frequently includes the use of corticosteroids and antihistamines but less frequently adrenaline, especially in Germany (19). The data from this study show a significant impact of an increased age and the higher usage rate of adrenaline among all severity grades of the reaction. This finding is striking considering the fact that the group of elderly patients suffered more frequently from cardiovascular diseases and took more concomitant medication (10). The higher usage of adrenaline in the elderly might also be due to the higher percentage of patients experiencing grade IV reactions, since adrenaline is often used in cardiopulmonary resuscitation. Even though adrenaline was applied more often in the elderly than in younger adults investigated here, still < 1 out of three patients received adrenaline as first line treatment. Adrenaline was mostly applied intravenously by professionals in the elderly (data not shown) not fulfilling the recommendations of the current guidelines for the management of anaphylaxis (12). The administration of intramuscular adrenaline is the route of first choice, because the risk of cardiovascular side effects is considerably lower (20).

This data indicate the need of educational measures regarding the acute treatment of anaphylaxis not only among patients but also among professionals. In particular, in emergency medical professionals the recognition of barriers against the use of adrenaline by an intramuscular route requires more attention. Recently, also guidelines from the resuscitation council are in favor of the first line intramuscular route and are an important step forward to change current clinical practice (21).

Finally, we observed increased hospital admission rates and treatment in ICU in elderly patients. Such an observation has been reported in previous studies from the US (9, 22). Most studies explain this phenomenon attributed to more severe reactions in elderly patients (8). However, our data suggest, that the hospital and the ICU admission rate was significantly higher in the elderly independent from the severity grade of anaphylaxis. Therefore, we propose that mostly due to comorbidities the patients are more often hospitalized, whether biphasic anaphylaxis occurs more frequently in the elderly patients is not known, but should be analyzed in more detail in future studies.

### Strength and Limitations

We report on a large group of elderly European anaphylactic patients covering a 10-year observational period. The web-based assessment tool for registration covers several aspects of an anaphylactic reaction in a standardized manner, including elicitors, symptoms, co-factors, and emergency treatment. The online questionnaire was consistently revised and piloted (6).

The data are limited to patient records and not representative for a population as only patients are registered when seen for an allergy assessment after a given reaction.

## Conclusion

In this large-scale description of anaphylaxis in patients aged 65 years and older, we report on characteristic age-dependent elicitors, comorbidities, symptoms, and emergency treatment. Features of anaphylaxis in patients aged 65 or more years differ from those of the younger adult group. The elderly are more likely to have anaphylaxis without skin symptoms which is associated with more difficulties to make diagnosis of anaphylaxis and more severe reactions. Moreover, the risk for insect venom anaphylaxis raises with age. The hospitalization and ICU admission rate were higher in elderly patients independent from the severity grade indicating that such patients require intensified medical intervention.

This study is one of the first comprehensive studies on patients ≥65 years who experienced anaphylaxis. It contributes to a better management and identification of specific medical needs in an aging population.

## Ethics Statement

The study was approved by the Ethics Committee at Charité-Universitätsmedizin Berlin (the coordinating center) and by the local Ethics Committees.

## Author Contributions

SA and SD-B acquired data and were responsible for the concept and design of the manuscript, for interpretation of the data, writing and final approval of the manuscript. SD-B and WF were responsible for the data analysis and interpretation. MB, GC, MF-R, TH, CP, IP-G, J-MR, EO, and KS acquired data, revised the manuscript critically for important intellectual content, and approved the final manuscript. MW and RT performed conceptualization of the study and were responsible for supervised interpretation of the data, writing, and final approval of the manuscript.

### Conflict of Interest Statement

The authors declare that the research was conducted in the absence of any commercial or financial relationships that could be construed as a potential conflict of interest.

## Abbreviations

ICU: Intensive Care Unit.
