# Supplementary figures and images for: Anaphylaxis in Elderly Patients—Data From the European Anaphylaxis Registry
Source: Front Immunol. 2019 Apr 24;10:750. doi: 10.3389/fimmu.2019.00750 (PMC6491699; doi:10.3389/fimmu.2019.00750)

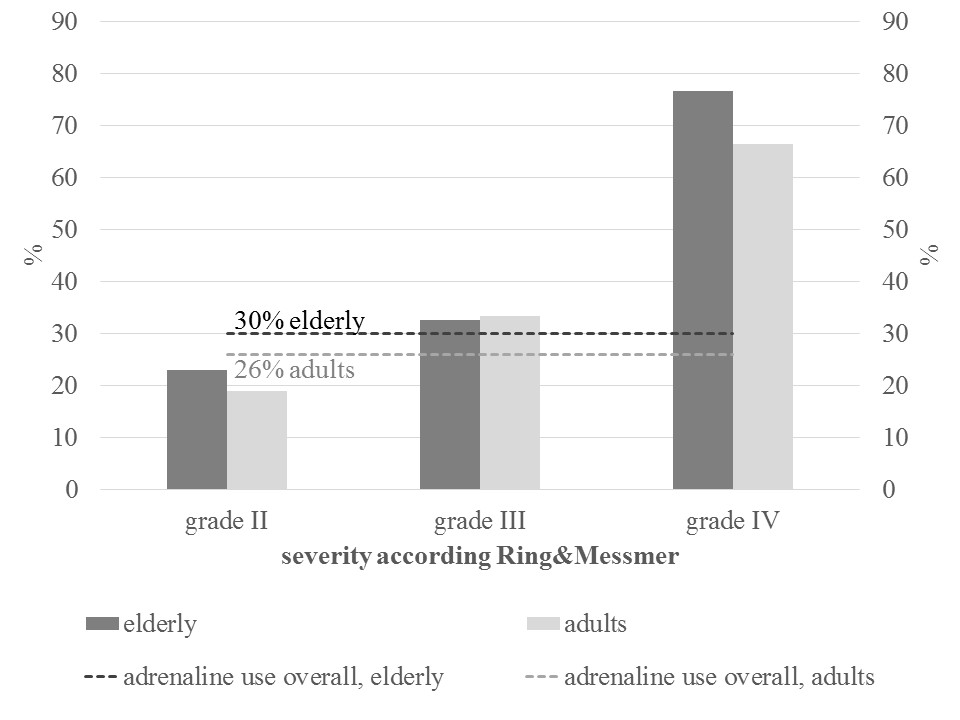


Figure S1: Adrenaline use by professionals in the first line.

Supplement: Supplementary file 1 [file Data_Sheet_1.docx]
